# Bulevirtide als erster spezifischer Wirkstoff gegen Hepatitis-D-Virusinfektionen – Mechanismus und klinische Wirkung

**DOI:** 10.1007/s00103-022-03486-2

**Published:** 2022-01-13

**Authors:** Shirin Nkongolo, Julius Hollnberger, Stephan Urban

**Affiliations:** 1 Molekulare Virologie, Translationale Virologie, Universitätsklinikum Heidelberg, Im Neuenheimer Feld 344, 69120 Heidelberg, Deutschland; 2 Deutsches Zentrum für Infektionsforschung (DZIF), Partnerstandort Heidelberg, Deutschland; 3 Toronto Centre for Liver Disease, University Health Network, Toronto, Kanada

**Keywords:** Hepatitis-D-Virus (HDV), Hepcludex®/Bulevirtide, Myrcludex B, Antivirale Therapie, Chronische virale Hepatitiden, Hepatitis D virus (HDV), Hepcludex®/bulevirtide, Myrcludex B, Antiviral therapy, Chronic viral hepatitis

## Abstract

Die Blockade des Zelleintritts von Krankheitserregern ist ein geeigneter Ansatz, um Neuinfektionen zu verhindern. Der therapeutische Einsatz von Eintrittsinhibitoren bei chronisch infizierten Patienten war jedoch bisher nur begrenzt erfolgreich. Zur Behandlung von chronischen Hepatitis-D-Virus-(HDV-)Infektionen wurde im Juli 2020 mit Bulevirtide (BLV) ein vielversprechender Wirkstoff bedingt zugelassen, der auf diesem Wirkprinzip beruht. Zuvor hatten für HDV keine gezielte Medikation zur Verfügung gestanden und die Behandlung beruhte auf dem Off-Label-Einsatz von Interferon-Alpha/Peginterferon-Alpha (IFNα/Peg-IFNα). In diesem Beitrag wird ein Überblick über die Grundlagen des Wirkmechanismus von BLV gegeben und bisher vorliegende klinische Daten werden zusammengefasst.

Eine HDV-Infektion manifestiert sich als Ko- oder Superinfektion bei Hepatitis-B-Virus-(HBV-)Infektionen und betrifft 4,5–15 % der HBV-Patienten weltweit. HDV nutzt die Hüllproteine von HBV zur Verbreitung. BLV wirkt, indem es den HBV/HDV-Rezeptor natriumtaurocholat-co-transportierendes Polypeptid (NTCP) blockiert und so den Eintritt von HBV/HDV in Hepatozyten verhindert. BLV senkt die HDV-Serum-RNA-Spiegel und führt bei HBV/HDV-infizierten Personen zur Normalisierung der Alanin-Aminotransferase-(ALT-)Werte. Es hat ein ausgezeichnetes Sicherheitsprofil, selbst wenn es über 48 Wochen in hohen Dosen (10 mg täglich) verabreicht wird. In Kombination mit Peg-IFNα zeigt BLV synergistische Effekte auf die Senkung der HDV-RNA im Serum, aber auch auf die Hepatitis-B-Oberflächenantigen-(HBsAg‑)Spiegel. Dies führte bei einer Untergruppe von Patienten zu einer funktionellen Heilung, wenn 2 mg BLV plus Peg-IFNα verabreicht wurden. Der Mechanismus dieser wahrscheinlich immunvermittelten Eliminierung wird in Folgestudien untersucht.

## Abstract (en)

Blocking the cell entry of pathogens is a suitable approach to prevent new infections. However, the therapeutic use of entry inhibitors in chronically infected patients has had limited success. For the treatment of chronic hepatitis D virus (HDV) infections, a promising agent based on this mode of action, Bulevirtide (BLV), was conditionally approved in July 2020. Previously, no drugs were available for HDV, and treatment relied on off-label use of interferon alpha/peginterferon alpha (IFNα/Peg-IFNα). In this review, we provide an overview of the basic mechanism of action of BLV and summarize the clinical data available to date.

HDV infection manifests as a co-infection or superinfection of hepatitis B virus (HBV) infections and affects 4.5–15% of HBV patients worldwide. HDV utilizes the envelope proteins of HBV for dissemination. BLV acts by blocking the HBV/HDV receptor sodium taurocholate co-transporting polypeptide (NTCP), preventing HBV/HDV entry into hepatocytes. BLV lowers HDV serum RNA levels and normalizes alanine aminotransferase (ALT) levels in HBV/HDV-infected individuals. It has an excellent safety profile, even when administered at high doses (10 mg daily) for 48 weeks. In combination with Peg-IFNα, BLV shows synergistic effects on lowering serum HDV RNA, but also on hepatitis B surface antigen (HBsAg) levels. This resulted in a functional cure in a subset of patients when 2 mg BLV plus Peg-IFNα was administered. The mechanism of this likely immune-mediated elimination will be investigated in follow-up studies.

## Einleitung

Hepatitis-B-Virus-(HBV-)Infektionen und Koinfektionen mit dem Hepatitis-D-Virus (HDV) verursachen chronische Lebererkrankungen, die zu einem deutlich erhöhten Risiko einer Leberzirrhose und eines hepatozellulären Karzinoms (HCC) führen [1]. Die Folgen der Infektion manifestieren sich häufig erst nach Jahrzehnten und die Krankheitslast der derzeit > 250 Mio. chronisch HBV-infizierten und mindestens 12 Mio. HDV/HBV-koinfizierten Personen ist hoch [2, 3]. Die antivirale Behandlung von HBV mit Nukleosid‑/Nukleotidanaloga (NUC) hemmt die vom Virus codierte reverse Transkriptase, verlangsamt die virale Replikation und das Fortschreiten der Krankheit, beseitigt aber nicht das persistierende Virusgenom in Hepatozyten, die cccDNA („covalently closed circular DNA“).

Die Koinfektion HBV-infizierter Personen mit HDV verschlechtert die klinische Prognose [4]. HDV ist ein viroidähnliches Satellitenvirus, das die HBV-Hüllproteine zur Verbreitung nutzt. Es unterdrückt die Replikation von HBV durch bisher unbekannte Mechanismen [5, 6]. Das beschleunigte Versagen der Leberfunktion ist unabhängig von HBV und wird auf HDV selbst zurückgeführt. Dazu passt die Beobachtung, dass die Unterdrückung von HBV durch Nucleos(t)id Analoga (NUC) bei HDV-Koinfizierten unwirksam ist [7, 8]. Interferon-Alpha (IFNα) wird als begrenzt wirksame Therapie gegen chronische HDV-Infektionen eingesetzt, jedoch mit potenziell erheblichen unerwünschten Wirkungen. Der Wirkstoff Bulevirtide (BLV) hemmt spezifisch den Eintritt von HBV und HDV in die Wirtszelle. Er ermöglicht die erste gezielte Therapie mit ausgeprägter antiviraler Wirkung gegen HDV bei sehr guter Verträglichkeit.

Dieser Artikel beinhaltet eine kurze Übersicht über bisher primär eingesetzte Therapien bei chronischer HBV/HDV-Infektion, konzentriert sich auf Grundlagen und Wirkmechanismus von BLV sowie Ergebnisse klinischer Studien mit diesem Wirkstoff, der seit Juli 2020 von der Europäischen Arzneimittelagentur (EMA) zur HBV/HDV-Therapie begrenzt zugelassen ist.

### Chronische HBV- und HDV-Infektionen und die derzeitigen Behandlungsmöglichkeiten

#### Die Hepatitis-B-Infektion

Etwa 2 Fünftel der Weltbevölkerung sind im Laufe ihres Lebens mit HBV in Kontakt gekommen. Davon haben mehr als 250 Mio. Menschen eine chronische Hepatitis B (CHB) entwickelt [9], die als ein anhaltender Nachweis von HBV-DNA und Hepatitis-B-Oberflächenantigen (HBsAg) im Serum für mehr als 6 Monate definiert ist. Die weltweite Prävalenz von HBsAg im Jahr 2016 wurde auf 3,9 % geschätzt [2]. CHB verursacht Leberzirrhose und Das hepatozälluläre Carcinom (HCC), was jährlich zu etwa 887.000 Todesfällen führt. Trotz der erfolgreichen Umsetzung von Impfprogrammen in vielen Ländern steigt die Zahl der Todesfälle durch CHB weiterhin an [9].

Eine akute HBV-Infektion heilt bei den meisten immunkompetenten Erwachsenen (90–95 %) aus. Im Gegensatz dazu kommt es bei Neugeborenen und Kleinkindern in > 90 % der Fälle zu einer Chronifizierung als Folge der perinatalen Übertragung des Virus [10]. Eine natürliche funktionelle Heilung der HBV-Infektion ist definiert als dauerhafte Negativierung der HBV-Serum-DNA und des HBsAg ohne therapeutische Intervention, begleitet von einer Serokonversion zu einem anti-HBsAg-positiven Immunstatus. Bemerkenswerterweise schließt dieser Status ein Wiederauftreten von HBV nicht aus (z. B. während immunsuppressiver Therapien nach einer Transplantation oder während einer Chemotherapie), was darauf hinweist, dass HBV-Genome (cccDNA) nicht vollständig eliminiert werden und reaktiviert werden können [11]. Eine *funktionelle Heilung *(„functional cure“) als Therapieziel ist dementsprechend definiert als HBV-DNA- und HBsAg-Negativität mit oder ohne Serokonversion zu Anti-HBsAg [12] nach antiviraler Behandlung. Bei chronisch infizierten Personen wird dies durch die derzeitigen Therapien mit NUC [13–15] fast nie und nach IFNα nur selten erreicht. Das Ziel aktueller klinischer Studien ist es, nicht nur die Virusreplikation zu unterdrücken, sondern eine funktionelle Heilung der CHB herbeizuführen.

Zur Behandlung der CHB sind derzeit nur NUC (z. B. Tenofovir und Entecavir) und IFNα/pegyliertes (Peg‑)IFNα zugelassen. Da NUC gut verträglich sind, aber nur selten zu einer HBsAg-Clearance und Serokonversion führen, werden sie häufig als Langzeit- oder lebenslange Therapie verabreicht. Durch die NUC-vermittelte Hemmung der HBV-Replikation werden i. d. R. die HBV-DNA im Serum verringert oder negativiert, die Alanin-Aminotransferase-(ALT-)Werte normalisiert und das Fortschreiten der Lebererkrankung verzögert oder verhindert [16, 17]. Im Vergleich zu NUC ist IFNα weniger wirksam in Hinblick auf den Rückgang der HBV-DNA, jedoch sind die Raten der HBsAg-Negativierung nach 48 Wochen Therapie höher, insbesondere bei Patienten mit niedrigen Ausgangswerten von HBsAg. Peg-IFNα wird i. d. R. bis zu 12 Monate lang unter strenger Überwachung angewendet, da es nur begrenzt verträglich ist und verschiedene potenziell ausgeprägte unerwünschte Arzneimittelwirkungen hat [18].

#### Die Hepatitis-D-Infektion

HDV ist ein RNA-Virus, welches die HBV-Hüllproteine zur Verbreitung seines viroidähnlichen Genoms benötigt. Eine HDV-Infektion manifestiert sich als Ko- oder Superinfektion von HBV-Trägern und verschlechtert deren Krankheitsprognose erheblich [9, 19]. Etwa 4,5–15 % der HBV-Patienten weltweit sind mit HDV koinfiziert. Dies könnte jedoch unterschätzt sein, da in vielen Ländern (einschließlich Industrieländern wie den USA oder China) bei chronisch HBV-infizierten Patienten nicht routinemäßig auf die HDV-Marker Anti-HDAg oder HDV-RNA getestet wird, obwohl dies in den meisten Leitlinien empfohlen wird. In einigen Ländern (z. B. in der Mongolei) weisen die Ergebnisse systematischer diagnostischer Screenings auf eine HDV-Prävalenz von > 60 % bei HBV-infizierten Patienten hin [9, 20].

Die Entwicklung von Medikamenten zur Behandlung chronischer Hepatitis D (CHD) ist (u. a. durch Limitationen geeigneter HDV/HBV-Infektionssysteme, Schwierigkeiten, die wirtszellabhängige Replikation zu adressieren, aber auch mangelndes Interesse von Pharmaunternehmen) erschwert. Da NUC weder direkt in die HDV-RNA-Replikation eingreifen noch HBsAg unterdrücken, welches für die Bildung und die Verbreitung von HDV-Partikeln erforderlich ist, werden sie als „Backbonetherapie“ zur HBV-Unterdrückung bei CHD-Patienten eingesetzt. Im Gegensatz dazu hat IFNα eine direkte antivirale Wirkung auf HDV, wie in Fallstudien und mehreren klinischen Studien gezeigt wurde. Es wird bisher als Off-Label-Therapie bei geeigneten Patienten eingesetzt. IFNα hat jedoch nur eine begrenzte Wirksamkeit hinsichtlich der HDV-Suppression und nach Therapieende kommt es häufig zu einem Wiederanstieg der Viruslast, in vielen Fällen sogar nach erfolgter Serum-HDV-RNA-Negativierung [21]. Daher werden dringend neue Therapien benötigt, um HBV/HDV-koinfizierte Patienten angemessen zu behandeln.

Das klinisch am weitesten entwickelte Medikament zur Behandlung von HDV/HBV-Koinfektionen ist BLV, früher als Myrcludex B (MyrB) bezeichnet. Daneben gibt es weitere Ansätze in der klinischen Entwicklung: Lonafarnib, ein Farnesyltransferaseinhibitor; Interferon-Lambda (IFNλ), ein Typ-III-IFN mit höherem Lebertropismus im Vergleich zu IFNα, und Rep2139, ein Oligonukleotid mit komplexen Wirkmechanismen. Ein Überblick über diese Moleküle findet sich in [22].

## Eintritt von HBV und HDV in Hepatozyten und Eintrittsblockade durch Bulevirtide (BLV)

### Eintrittsmechanismus von HBV/HDV

**Figure 1:**
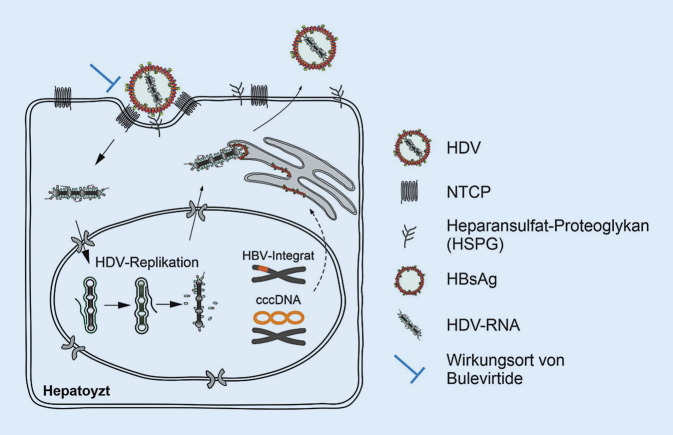

HDV nutzt die Hüllproteine von HBV sowohl für den Austritt aus infizierten Hepatozyten als auch für den Eintritt (*De-novo*-Eintritt) in rezeptorexprimierende uninfizierte Zellen, in bereits HDV-infizierte Hepatozyten oder in Zellen, die den Rezeptor exprimieren, aber auch (integrierte) HBV-DNA tragen. Für diesen rezeptorvermittelten Eintritt von HBV und HDV ist der Gallensalztransporter natriumtaurocholat-co-transportierendes Polypeptid (NTCP) erforderlich ([23, 24]; Abb. 1). Nach initialer Bindung der HBV-Hüllproteine an Heparansulfat-Proteoglykane unter Beteiligung der PreS-Domäne und des „antigenic loop“ der S‑Domäne [25, 26] bindet die myristoylierte PreS1-Domäne des großen (L-)HBV-Oberflächenproteins NTCP mit hoher Affinität [27]. Dies ermöglicht die Aufnahme und anschließende Fusion der viralen und zellulären Membranen.

### Hemmung des Viruseintritts durch BLV

Systematische Untersuchungen zur HBV-Infektionshemmung unter Verwendung synthetischer Lipopeptide, die die N‑terminale PreS1-Domäne des HBV-L-Proteins nachahmen [28–30], waren ein wichtiger Ansatz sowohl für die Identifizierung des HBV-Rezeptors als auch für die Entwicklung von BLV als klinisch anwendbarem Eintrittsinhibitor [31]. (Für einen Überblick über die Ansätze, die zur Identifizierung viraler Infektionsdeterminanten, zur Identifizierung des Rezeptors und zur Entwicklung von BLV führten, siehe [27].)

BLV ist ein optimiertes Peptid, das von einer verkürzten Konsensussequenz der HBV-Hüllproteine abgeleitet ist. Es hemmt die Infektion menschlicher Hepatozyten (PHH) mit HBV und HDV bereits in pikomolaren Konzentrationen (IC50 = 80 pM). In PHH wird der Viruseintritt bereits mit 30 nM komplett blockiert, was etwa der 10fachen IC90 (IC90 = 3 nM; 16 ng/ml) entspricht. Pharmakokinetische Studien an gesunden Freiwilligen zeigten Serumkonzentrationen oberhalb der IC90 für ca. 14 h nach einer einmaligen subkutanen (s. c.) Bolusinjektion von 10 mg BLV [32].

NTCP wird ausschließlich an der sinusoidalen Seite von Hepatozyten exprimiert [33]. Wahrscheinlich ist diese exklusive Expression des Rezeptors die Hauptdeterminante des Hepatotropismus von HBV und HDV. Dementsprechend bindet BLV nach systemischer Verabreichung spezifisch an die parenchymatösen Leberzellen, was durch *In-vitro-*Studien und in Tiermodellen (Mäuse, Ratten, Hunde, Schimpansen), die ein bindungskompetentes Homolog von NTCP exprimieren, nachgewiesen wurde. Nach der Bindung an NTCP verbleibt BLV an der Plasmamembran von Hepatozyten mit einer außergewöhnlich langen Halbwertszeit von etwa 12–18 h [34, 35].

Die Inaktivierung der NTCP-Rezeptorfunktion durch BLV ist höchstwahrscheinlich ein irreversibler Prozess. Folglich erfordern mit BLV behandelte Hepatozyten vermutlich eine *De-novo-*NTCP-Synthese und den Transport zur Plasmamembran sowie den Umsatz/Abbau von inaktivierten NTCP/BLV-Komplexen, um erneut für HBV/HDV empfänglich zu sein. NTCP-Substrate wie Ezetimib und Gallensäuren hemmen ebenfalls die HBV-Infektion [24, 36, 37]. Da sie als Substrate von NTCP nur für eine sehr kurze Zeit reversibel binden und dann in das Zytoplasma der Hepatozyten transportiert werden, sind sie sehr wahrscheinlich als antivirale Medikamente nicht geeignet.

### Hemmung anderer NTCP-Funktionen durch BLV

BLV stört die natürliche Funktion von NTCP, nämlich die (Wieder‑)Aufnahme von konjugierten Gallensalzen wie Cholsäure (CA), Ursodeoxycholsäure (UDCA) und anderen natürlichen Substraten in die Leber im Rahmen des enterohepatischen Kreislaufs [24, 36]. Daneben werden einige Hormone, Medikamente wie Statine, Xenobiotika und andere Moleküle von NTCP transportiert [38]. Daher ist zu erwarten, dass die Pharmakokinetik und der metabolische Umsatz dieser Substanzen unter einer hoch dosierten BLV-Therapie (bzw. NTCP-sättigenden Dosen von > 3 mg/Patient) beeinflusst werden. Für Patienten, die sowohl mit BLV als auch mit anderen von NTCP transportierten Arzneimitteln behandelt werden, wird eine Substitution durch Medikamente einer anderen Wirkstoffklasse empfohlen.

Bemerkenswert ist, dass die Hemmung der Gallensalzaufnahme höhere BLV-Konzentrationen (IC50 = 50–150 nM, je nach Assay) erfordert als die Hemmung des Viruseintritts. Diese IC50-Werte entsprechen den Konzentrationen, die zu einer halbgesättigten Bindung von NTCP führen, was darauf hindeutet, dass die stöchiometrische Besetzung der NTCP-Bindungsstellen mit BLV mit der Störung des Gallensalztransports korreliert. Der Unterschied in der Wirksamkeit hinsichtlich der Hemmung des HBV/HDV-Eintritts und der Hemmung des Gallensalz‑/Substrattransports könnte damit zusammenhängen, dass NTCP oligomere Komplexe bildet [39]. Während jede Untereinheit das natürliche Substrat transportieren kann, erfolgt die Aufnahme von HBV und HDV nur, wenn mehrere Wechselwirkungen des HBV-L-Proteins mit NTCP gebildet werden, und oligomere Komplexe sind somit notwendig, um Viruseintritt zu vermitteln. Die Blockade nur einer Untereinheit kann daher die Infektion verhindern, ermöglicht aber weiterhin den Transport von Gallensalzen und anderen Substraten.

### Weitere Auswirkungen der BLV-Behandlung auf zellulärer Ebene

Die ausgeprägte Spezifität von BLV für NTCP ist bemerkenswert und spiegelt die evolutionäre Anpassung der Hüllproteine von Primatenhepadnaviren an den hepatozytenspezifischen Rezeptor ihrer jeweiligen Wirte wider. In sehr hohen Konzentrationen interferiert BLV jedoch auch mit 2 anderen Mitgliedern der ABC-Transporter-Familie, OATP1B1 und OATB1B3 (IC50 500 nM bzw. 8700 nM; [40]). Diese Wirkung ist von demselben hoch konservierten PreS-Sequenzmotiv innerhalb des Peptids (9-NPLGFFP-15) abhängig wie die NTCP-Bindung, was die evolutionäre Verwandtschaft dieser Transporter mit der ABC-Transporterfamilie verdeutlicht. Die bei der klinischen Anwendung von BLV für HBV/HDV-Infektionen verwendeten Dosierungen (2–10 mg täglich) führen jedoch zu Plasmakonzentrationen, die die Funktion anderer ABC-Transporter als NTCP nicht blockieren.

Entsprechend seines Wirkmechanismus beeinträchtigt BLV die HBV- und HDV-Replikation in bereits infizierten Zellen nicht. Seine antivirale Wirksamkeit hängt daher von der Stabilität und Umsatzdynamik der HBV/HDV-Episome in bereits infizierten Hepatozyten und von deren Halbwertszeiten ab. Letztere sind nicht genau bekannt, aber vermutlich deutlich kürzer als jene von nicht infizierten Zellen. Vermutlich hängt die antivirale Wirkung von BLV auch von der immunvermittelten Zytolyse bei Leberentzündung ab. Dementsprechend ist ein Synergismus von BLV mit Immunmodulatoren wie IFN, die die zytolytische Abtötung fördern, zu erwarten.

Ein sekundärer Wirkmechanismus könnte zudem mit der Modulation der Wirts- und Virusgenexpression durch eine gallensäurenabhängige (vom Farnesoid-X-Rezeptor abhängige) Transkriptionskontrolle zusammenhängen [41, 42]. Ein möglicher Beitrag dieses Effekts zur antiviralen Aktivität von BLV sollte in Zukunft untersucht werden.

## Erkenntnisse aus klinischen Studien mit BLV

### Ausgezeichnete Verträglichkeit von BLV in klinischer Phase-I-Studie (MYR101)

BLV kann sowohl intravenös (i.v.) als auch s.c. verabreicht werden. Eine Phase-I-Sicherheitsstudie wurde 2011 durchgeführt [32]: 36 gesunde Probanden erhielten einmalig Dosierungen bis zu 20 mg BLV i.v. oder bis zu 10 mg s.c. Die Substanz wurde sehr gut vertragen, ohne schwerwiegende unerwünschte Ereignisse („serious adverse reactions“ – SAR) zu induzieren. Es gab keine Anzeichen für Off-Target-Effekte oder Immunogenität. Bemerkenswert ist, dass dies selbst bei einer i.v.-Gabe von 20 mg der Fall war – diese Dosis liegt über dem Bereich von 0,5–10 mg, der ausreicht, um bei Patienten eine effektive antivirale Wirkung zu erzielen.

Die Pharmakokinetik folgte einem 2‑Kompartiment-Dispositionsmodell und deutet darauf hin, dass BLV zunächst die hoch affinen NTCP-Bindungsstellen in der Leber sättigt und anschließend der Kinetik seines Komplexes mit Serumalbumin folgt. Die Bioverfügbarkeit nach s.c.-Injektion wurde auf 85 % geschätzt. In den folgenden Studien wurde BLV s.c. verabreicht. Eine bereits präklinisch entwickelte Formulierung für die orale Gabe muss noch weiter verbessert werden, um ausreichende Mengen oral verabreichen zu können [43].

### Phase-II-Studien zur BLV-Therapie in Kombination mit Tenofovir (MYR202) oder Peg-IFNα (MYR203)

Die erste Phase-II-Studie mit einem HDV-spezifischen primären Endpunkt ist MYR202 [44, 45]. Hier wurden 120 Patienten zunächst für mindestens 12 Wochen mit 245 mg Tenofovir (TDF) täglich behandelt und anschließend über 24 Wochen täglich mit TDF allein oder plus 2 mg, 5 mg oder 10 mg BLV therapiert. Alle Patienten hatten keine oder nur geringgradige Leberzirrhose (Child-Pugh-Score ≤ 6) und kein HCC in der Krankheitsgeschichte (Tab. 1). Den primären Endpunkt – Negativierung von Serum-HDV-RNA (HDV-RNA < LLOD, „lower limit of detection“) oder Reduktion > 2 log_10_ – erreichten 60 % der BLV-Patienten (2 mg, 5 mg oder 10 mg), jedoch nur 3,6 % der Patienten ohne BLV-Therapie (Tab. 2). Bemerkenswerterweise zeigten gepaarte Leberbiopsien eine Reduktion der Zahl HDV-infizierter Hepatozyten unter BLV-Therapie, korrelierend mit Reduktion der intrahepatischen HDV-RNA und Serum-HDV-RNA [46]. Ein mathematisches Modell für Serum-HDV-RNA ergab eine Kinetik nullter Ordnung, nach der HDV in den meisten Patienten nach 2–3 Jahren Behandlung eliminiert werden könnte. 40–50 % der BLV-behandelten Patienten normalisierten eine zuvor erhöhte ALT bis Woche 24 gegenüber 6,6 % der Patienten ohne BLV-Therapie. Die kombinierte therapeutische Antwort aus HDV-Reduktion und ALT-Normalisierung kam nur bei BLV-behandelten Patienten vor, und zwar bei > 20 % in jeder dieser 3 Gruppen (Tab. 3). Die Ergebnisse zeigten eine bedingte Dosisabhängigkeit von BLV bezüglich des antiviralen Effekts (HDV-RNA), jedoch nicht des biochemischen Effekts (ALT). Nach Ende der Therapie kam es zu einem Wiederanstieg der HDV-RNA und der ALT-Werte. Nur 3–7 % der BLV-behandelten Patienten konnten ihre kombinierte Therapieantwort 24 Wochen nach Therapieende aufrechterhalten.

**Table 1:** 

Klinische Studien (Identifikator)	MYR202 (NCT03546621)	MYR203 (NCT02888106)	MYR204 (NCT03852433)	MYR301 (NCT03852719)
*Wichtigste Einschlusskriterien*	18–65 Jahre	18–65 Jahre	18–65 Jahre	18–65 Jahre
	Positive Serum-HDV-RNA (PCR)	Positive Serum-HDV-RNA (PCR)	Positive Serum-HDV-RNA (PCR)	Positive Serum-HDV-RNA (PCR)
	ALT ≥ 1 × ULN und < 10 × ULN	ALT ≥ 1 × ULN und < 10 × ULN	ALT ≥ 1 × ULN und < 10 × ULN	ALT ≥ 1 × ULN und < 10 × ULN
	–	–	Serumalbumin > 28 g/L	Serumalbumin > 28 g/L
*Wichtigste Ausschlusskriterien*	**Child-Pugh**-Score > **6 Punkte**	**Child-Pugh**-Score **≥** **6 Punkte**	**Child-Pugh**-Score **>** **6 Punkte**	**Child-Pugh**-Score **>** **7 Punkte**
	–	Thrombozyten < 90.000/µL	Thrombozyten < 90.000/µL	Thrombozyten 60.000/µL
	–	Hämoglobin < 10 g/dL	Hämoglobin < 12 g/dL	–
	HCV^a^- oder HIV-Koinfektion	HCV^a^- oder HIV-Koinfektion	HCV^a^- oder HIV-Koinfektion	HCV^a^- oder HIV^b^-Koinfektion
	Kreatinin-Clearance < 60 mL/min	Serumkreatinin > 1,5 × ULN	Kreatinin-Clearance < 60 mL/min	Kreatinin-Clearance < 60 mL/min
	Gesamtbilirubin ≥ 34,2 µmol/L	Gesamtbilirubin ≥ 34,2 µmol/L	Gesamtbilirubin ≥ 34,2 µmol/L	Gesamtbilirubin ≥ 34,2 µmol/L
	Frühere oder aktuelle maligne Neoplasien, einschließlich Leberzellkarzinom (**HCC**)	Frühere oder aktuelle maligne Neoplasien, einschließlich Leberzellkarzinom (**HCC**)	Frühere oder aktuelle maligne Neoplasien, einschließlich Leberzellkarzinom (**HCC**)	Frühere oder aktuelle maligne Neoplasien, einschließlich Leberzellkarzinom (**HCC**)

*HDV* Hepatitis-D-Virus, *ALT* Alanin-Aminotransferase, *ULN* „upper limit of normal“, *HCV* Hepatitis-C-Virus, *HIV* humanes Immundefizienzvirus, *HCC* hepatozelluläres Karzinom

^a^Probanden mit Anti-HCV-Antikörpern konnten in die Studie aufgenommen werden, wenn der HCV-RNA-Screeningtest negativ war

^b^Probanden mit einer HIV-Infektion konnten aufgenommen werden, wenn die Anzahl der CD4+ > 500/ml betrug und die HIV-RNA mindestens 12 Monate lang unter der Nachweisgrenze lag

**Table 2:** 

		Patientenanzahl	Therapiedauer (Wochen)	HDV-RNA-Negativierung (HDV-RNA < LLOD)			HDV-RNA > 2 log_10_ Reduktion oder Negativierung			HBsAg > 1 log_10_ IU/ml Reduktion oder Negativierung		
				Unter Therapie		Nach Therapie	Unter Therapie		Nach Therapie	Unter Therapie		Nach Therapie
Klinische Studien (Identifikator)	Therapiearme	Baseline		Woche 24 (in %)	Woche 48 (in %)	Woche 24 (in %)	Woche 24 (in %)	Woche 48 (in %)	Woche 24 (in %)	Woche 24 (in %)	Woche 48 (in %)	Woche 24 (in %)
*MYR202 (NCT03546621)*	BLV 2 mg QD + TDF	28	24	–	–	–	53,6	–	7,1	–	–	–
	BLV 5 mg QD + TDF	32	24	–	–	–	50,0	–	3,1	–	–	–
	BLV 10 mg QD + TDF	30	24	–	–	–	76,7	–	10,0	–	–	–
	TDF	28	24	–	–	–	3,6	–	0,0	–	–	–
*MYR203 (NCT02888106)*	Peg-IFNα 180 μg QW	15	48	6,7	13,3	0,0	–	–	0,0	6,7	0,0	0,0
	BLV 2 mg QD	15	48	13,3	13,3	6,7	–	–	33,3	0,0	0,0	0,0
	BLV 10 mg QD + TDF	15	48	26,7	46,7	33,3	–	–	46,7	6,7	0,0	0,0
	BLV 2 mg QD + Peg-IFNα 180 μg QW	15	48	60,0	80,0	53,3	–	–	73,3	40,0	46,7	40,0
	BLV 5 mg QD + Peg-IFNα 180 μg QW	15	48	60,0	86,7	26,7	–	–	–	13,3	20,0	13,3
	BLV 10 mg QD + Peg-IFNα 180 μg QW	15	48	67,7	80,0	6,7	–	–	33,3	6,7	6,7	13,3
*MYR204 (NCT03852433)*	Peg-INFα 180 µg QW	25	48	13,0	–	–	37,5	–	–	4,2	–	–
	BLV 2 mg QD + Peg-IFNα 180 µg QW, dann BLV 2 mg QD	50	48 + 48	24,0	–	–	88,0	–	–	12,0	–	–
	BLV 10 mg QD + Peg-INFα 180 µg QW, dann BLV 10 mg QD	50	48 + 48	34,0	–	–	92,0	–	–	8,0	–	–
	BLV 10 mg QD	50	96	4,0	–	–	72,0	–	–	0,0	–	–
*MYR301 (NCT03852719)*	BLV 2 mg QD	50	144	6,0	–	–	55,1	–	–	2,0	–	–
	BLV 10 mg QD	50	144	8,0	–	–	68,0	–	–	0,0	–	–
	BLV 10 mg QD^b^	50	96	0,0	–	–	4,0	–	–	0,0	–	–

*HDV* Hepatitis-D-Virus, *LLOD* „lower limit of detection“ (untere Nachweisgrenze), *HBsAg* Hepatitis-B-Oberflächenprotein, *BLV* Bulevirtide, *QD* einmal pro Tag, *TDF* Tenofovir, *Peg-INFα* pegyliertes Interferon‑α, *QW* einmal pro Woche

^a^Tenofovir-(TDF-)vorbehandelt

^b^Therapiearm startet verzögert nach einem Beobachtungszeitraum von Woche 48

**Table 3:** 

		Patientenanzahl		ALT-Normalisierung			Kombinierte Antwort^c^		
				Unter Therapie		Nach Therapie	Unter Therapie		Nach Therapie
Klinische Studien (Identifikator)	Therapiearme	Baseline	Therapiedauer (Wochen)	Woche 24 (in %)	Woche 48 (in %)	Woche 24 (in %)	Woche 24 (in %)	Woche 48 (in %)	Woche 24 (in %)
*MYR202 (NCT03546621)*	BLV 2 mg QD + TDF	28	24	42,8	–	–	21,4	–	7,1
	BLV 5 mg QD + TDF	32	24	50,0	–	–	28,1	–	3,1
	BLV 10 mg QD + TDF	30	24	40,0	–	–	36,7	–	3,3
	TDF	28	24	6,6	–	–	0,0	–	0,0
*MYR203 (NCT02888106)*	Peg-IFNα 180 μg QW	15	48	0,0	26,7	10,0	0,0	6,7	0,0
	BLV 2 mg QD	15	48	64,3	73,3	23,1	13,3	13,3	6,7
	BLV 10 mg QD + TDF	15	48	60,0	40,0	35,7	13,3	13,3	13,3
	BLV 2 mg QD + Peg-IFNα 180 μg QW	15	48	6,7	26,7	53,8	6,7	20,0	46,7
	BLV 5 mg QD + Peg-IFNα 180 μg QW	15	48	20,0	46,7	33,3	20,0	33,3	13,3
	BLV 10 mg QD + Peg-IFNα 180 μg QW	15	48	20,0	26,7	35,7	13,3	20,0	6,7
*MYR204 (NCT03852433)*	Peg-INFα 180 µg QW	25	48	12,5	–	–	12,5	–	–
	BLV 2 mg QD + Peg-IFNα 180 µg QW, dann BLV 2 mg QD	50	48 + 48	30,0	–	–	30,0	–	–
	BLV 10 mg QD + Peg-INFα 180 µg QW, dann BLV 10 mg QD	50	48 + 48	24,0	–	–	24,0	–	–
	BLV 10 mg QD	50	96	64,0	–	–	50,0	–	–
*MYR301 (NCT03852719)*	BLV 2 mg QD	50	144	53,1	–	–	36,7	–	–
	BLV 10 mg QD	50	144	38,0	–	–	28,0	–	–
	BLV 10 mg QD^b^	50	96	6,0	–	–	0,0	–	–

*ALT* Alanin-Aminotransferase, *BLV* Bulevirtide, *QD* einmal pro Tag, *TDF* Tenofovir, *Peg-INFα* pegyliertes Interferon‑α, *QW* einmal pro Woche

^a^Tenofovir-(TDF-)vorbehandelt

^b^Therapiearm startet verzögert, nach einem Beobachtungszeitraum von Woche 48

^c^Kombinierte Antwort: ALT-Normalisierung und HDV-RNA-Negativierung (MYR203) bzw. HDV-RNA > 2 log_10_ Reduktion (MYR204, MYR301)

In der MYR203-Studie [47, 48] wurde die Therapiedauer auf 48 Wochen ausgedehnt sowie ein möglicher Synergismus mit IFNα untersucht. Hierbei wurden je 15 Patienten in 4 Gruppen behandelt: IFNα (einmal wöchentlich: 180 µg), BLV (täglich: 2 mg) und BLV in Kombination mit IFNα (2 mg BLV + IFNα, 5 mg BLV + IFNα). In einer Erweiterung wurden 2 hoch dosierte Behandlungsgruppen hinzugefügt: 10 mg BLV + IFNα und 10 mg BLV + TDF. Eingeschlossen wurden Patienten ohne oder mit geringgradiger Leberzirrhose (Child-Pugh-Score ≤ 5; Hb > 10 g/dl) und ohne Historie eines malignen Geschehens (Tab. 1). Der primäre Endpunkt der Studie – Negativierung von HDV-RNA in Woche 72 (24 Wochen nach Therapieende) – wurde von 0 %, 6,7 %, 53,3 %, 26,7 %, 6,7 % und 33,3 % der Patienten der jeweiligen Arme erreicht (Tab. 2). Longitudinale Leberbiopsien von Patienten vor und nach Therapie mit BLV zeigten zudem erneut eine deutliche Reduktion der HDV-infizierten Hepatozyten sowie eine Reduktion der Nekroinflammation und Fibrose. Eine ALT-Normalisierung 24 Wochen nach Therapieende erreichten 10,0 %, 23,1 %, 53,8 %, 33,3 %, 35,7 % und 35,7 % der Patienten (Tab. 3). HBsAg-Reduktion > 1 log IU/ml bzw. HBsAg-Negativierung 24 Wochen nach Therapieende erreichten 40,0 %, 13,3 % und 13,3 % der Patienten, die eine Kombination von BLV und IFNα erhielten (2 mg BLV, 5 mg BLV, 10 mg BLV). Interessanterweise hatte BLV den stärksten Synergismus mit IFNα in der niedrigsten Dosierung (2 mg), sowohl hinsichtlich der virologischen Parameter (Reduktion von HDV-RNA und HBsAg) als auch hinsichtlich der ALT als Marker für Leberzellschaden.

### Aktuelle Phase-II- und Phase-III-Studien (MYR204 und MYR301)

Seit 2019 laufen 2 Studien mit BLV, welche die optimale Therapiedauer für BLV-Monotherapie und die Kombination mit IFNα untersuchen. In der erweiterten Phase-II-Studie MYR204 [49, 50] werden 175 Patienten wie folgt behandelt: 48 Wochen wöchentlich 180 µg IFNα; 48 Wochen 2 mg BLV + IFNα und 48 Wochen 2 mg BLV; 48 Wochen 10 mg BLV + IFNα und 48 Wochen 10 mg BLV; 96 Wochen 10 mg BLV. Die Phase-III-Studie MYR301 [51, 52] untersucht insgesamt 150 Patienten mit BLV-Monotherapie über einen Zeitraum von 96 Wochen (10 mg BLV) und 144 Wochen (2 mg und 10 mg BLV). Die Einschlusskriterien der MYR204-Studie (Child-Pugh-Score ≤ 6; Thrombozyten ≥ 90.000/µl) waren strenger als in der MYR301-Studie (Child-Pugh-Score ≤ 7; Thrombozyten ≥ 60.000/µl), sodass das Patientenkollektiv der MYR301 etwas weiter fortgeschrittene Verläufe beinhalten könnte. Erneut wurden in beiden Studien Patienten mit Historie eines HCC ausgeschlossen (Tab. 1).

Erste Zwischenergebnisse 24 Wochen nach Studienbeginn unterstreichen den dosisabhängigen Effekt von BLV auf die HDV-RNA-Reduktion sowie die Überlegenheit gegenüber IFNα-Monotherapie (Tab. 2). Ein Effekt auf HBsAg ist in Kombination mit IFNα sichtbar, was die Ergebnisse früherer Studien stützt. BLV-Monotherapie führt zu einer vermehrten ALT-Normalisierung, verglichen mit Kombinationstherapie mit IFNα (Tab. 3). Es ist jedoch noch unklar, wie lange dieser Effekt nach Ende der Therapie anhalten wird.

3 Patienten mit kompensierter Leberzirrhose (Child A) wurde BLV als *Compassionate-Use*-Therapie (Einsatz bisher nicht zugelassener Arzneimittel) über bis zu 3 Jahre verabreicht [53]. In 3 Fällen stabilisierte sich die Leberfunktion. Bei einem Patienten mit Ösophagusvarizen und portaler Hypertension bildeten sich die Varizen zurück und es wurde eine klinische Besserung erreicht.

## Weitere Studien zu Sicherheit und unerwünschten Wirkungen von BLV

BLV bindet selektiv an den Gallensalztransporter NTCP, der ausschließlich in Hepatozyten vorkommt. Dementsprechend ist eine sehr spezifische Aktivität zu erwarten und in der Tat haben die ersten Studien keine Off-Target-Effekte gezeigt. Die spezifische Bindung von BLV kann die natürliche Gallensalztransportfunktion von NTCP beeinträchtigen, für die *in vitro *eine IC50 von etwa 50 nM ermittelt wurde ([24, 36]; IC50 für die Beeinträchtigung des HBV/HDV-Eintritts: etwa 80 pM [31], was auf ein großes therapeutisches Fenster schließen lässt). Beim Menschen führen Dosierungen > 15 µg/kg (1 mg/Patient) zu ansteigenden Gallensäurespiegeln, wobei eine NTCP-Sättigung bei > 75 µg/kg (5 mg/Patient) auftritt.

In einer Sicherheitsstudie zu den Auswirkungen von BLV auf die Gallensäuredisposition und den pharmakokinetischen Wechselwirkungen mit Tenofovir erhielten 12 gesunde Freiwillige 300 mg Tenofovir und 10 mg BLV [40]. Die Plasmaspiegel der unkonjugierten Gallensäuren waren unter der BLV-Behandlung > 19fach erhöht, die der konjugierten Gallensalze bis zu 124fach. Dieser Unterschied lässt sich durch die höhere Transporterspezifität konjugierter Gallensalze erklären, die hauptsächlich auf NTCP angewiesen sind, im Vergleich zu nichtkonjugierten Gallensalzen, die auch von anderen Membranproteinen wie Mitgliedern der OATP-Familie transportiert werden können. Erhöhte Gallensäurespiegel blieben bei allen Probanden klinisch asymptomatisch. Es traten keine signifikanten Veränderungen in der Pharmakokinetik von Tenofovir auf.

In allen bisherigen Studien wurden die Gallensäurespiegel überwacht, was insbesondere im Hinblick auf eine verlängerte Behandlung von Bedeutung ist. Der größte Datensatz liegt für die Studie MYR202 vor, in der die Kombination von BLV (0 mg; 2 mg; 5 mg; 10 mg täglich) und Tenofovir untersucht wurde. Analysiert wurden nichtkonjugierte (Cholsäure) und konjugierte (z. B. Taurocholsäure und Glycocholsäure) Gallensalze. Während 12 Wochen BLV-Behandlung waren die Gallensäuren dosisabhängig angestiegen, mit einem bis zu 10,3fachen Anstieg für unkonjugierte und einem bis zu > 20fachen (5 mg BLV) bzw. > 8fachen (10 mg BLV) Anstieg für konjugierte Gallensalze. Dementsprechend waren auch die Gallensäuren im Urin erhöht, was auf eine Kompensation der Exkretion über die Harnwege hindeutet. Die Erhöhung der Gallensäuren war reversibel und erreichte eine Woche nach Behandlungsende wieder die Ausgangswerte.

Alle Fälle von Gallensäureerhöhungen im Zusammenhang mit der BLV-Behandlung waren klinisch asymptomatisch; insbesondere wurden kein Bilirubinanstieg und kein Juckreiz beobachtet. Passend dazu gibt es einen ersten beschriebenen Fall eines homozygoten NTCP-Polymorphismus, der zu einem funktionellen Knock-out des Transporters führte [54]. Trotz einer > 100fachen Erhöhung der Gallensäuren waren keine signifikanten klinischen Symptome beobachtbar.

In den bisherigen BLV-Studien traten während der Behandlung keine weiteren ernsthaften unerwünschten Ereignisse („severe adverse events“; SAE) auf. Nach Behandlungsende kam es bei 2 Patienten in der MYR202-Studie zu einer Hepatitisexazerbation. Dieses Wiederaufflammen (Flare) ist höchstwahrscheinlich auf eine virale Reaktion (Rebound) zurückzuführen und war selbstlimitierend ohne Intervention. Weitere unerwünschte Ereignisse waren vor allem Reaktionen an der Injektionsstelle nach S.-c.-Verabreichung von BLV (< 5 % der Patienten).

## Fazit

BLV ist ein synthetisches Lipopeptid, das von der myristoylierten PreS-Subdomäne des HBV-L-Proteins abgeleitet ist. Es blockiert sehr effizient den Eintritt von HBV/HDV in Hepatozyten durch spezifische Bindung an den Gallensalztransporter NTCP. Dieser fungiert als Komponente des enterohepatischen Kreislaufs der Gallensalze und ist der Rezeptor für den Eintritt von Primatenhepadnaviren.

Klinische Studien mit BLV zeigten sowohl ein günstiges Sicherheitsprofil als auch eine ausgeprägte antivirale Aktivität gegen HBV und HDV. Die antivirale Wirkung auf die Viruslast im Serum war gegen HDV ausgeprägter als gegen HBV. BLV reduzierte ALT- und Fibrosewerte auch in zirrhotischen Patienten. Entsprechend seines Wirkmechanismus bindet BLV schnell und effizient an NTCP und führte zu einer reversiblen Erhöhung der Plasmaspiegel von Gallensalzen. Bislang wurden keine Anzeichen für eine Tachyphylaxie und keine klinischen Symptome im Zusammenhang mit erhöhten Serumgallensalzspiegeln beobachtet. Eine BLV-Monotherapie oder eine Kombination mit TDF über einen Zeitraum von bis zu 24 Wochen führte nicht zu einer signifikanten Senkung der HBsAg-Spiegel, jedoch zu einer deutlichen Verringerung der HDV-RNA-Spiegel im Serum und in der Leber, begleitet von einer Reduktion HDV-infizierter Hepatozyten. Da BLV ausschließlich den *De-novo*-Eintritt von HDV verhindert, werden synergistische Effekte mit Medikamenten erwartet, die in die durch Zellteilung vermittelte HDV-Verbreitung eingreifen. Dieser zusätzliche Modus der HDV-Eliminierung wurde kürzlich beschrieben und es konnte gezeigt werden, dass IFN die durch Zellteilung vermittelte Ausbreitung von HDV-RNA unterdrücken [55]. Klinisch zeigte die kombinierte Behandlung von Patienten mit BLV und Peg-IFNα starke synergistische Effekte mit schneller HDV-RNA-Negativierung, selbst bei Patienten, bei denen die HBsAg-Werte nicht signifikant sanken. Eine Untergruppe der Patienten, die 2 mg BLV und Peg-IFNα erhielten, erreichte aber auch HBsAg-Verlust und HBsAg-Serokonversion. Der Mechanismus dieser wahrscheinlich immunvermittelten Eliminierung von HBsAg-produzierenden Zellen ist derzeit unklar und wird in Folgestudien untersucht.

Wie bei einem Eintrittsinhibitor zu erwarten, führte die IFN-freie Therapie mit BLV (mit oder ohne TDF) zu einer Reduktion der HDV-RNA mit einer Kinetik nullter Ordnung. Diese Kinetik deutet darauf hin, dass eine längere Therapie mit BLV HDV-RNA eliminieren könnte. Nach mathematischen Modellen sollten mehr als 50 % der Patienten nach > 2 Jahren unter hoch dosiertem BLV das Virus eliminiert haben, nach 3 Jahren sogar mehr als 90 %.

Es ist zu beachten, dass alle bisherigen Studien nur mit Patienten ohne oder mit geringgradiger Leberzirrhose, ohne Historie eines HCC und mit maximaler ALT ≤ 10 ULN durchgeführt wurden, sodass keine Daten zu Effekten bei höhergradiger Zirrhose und zum Verlauf bei HCC verfügbar sind.

Am 31.07.2020 wurde von der Europäischen Arzneimittelagentur (EMA) BLV, ein Präparat für die Behandlung von HDV/HBV-koinfizierten Erwachsenen mit kompensierter Lebererkrankung bedingt zugelassen. Die bedingte Zulassung erfolgte im Anschluss an die Ausweisung als Arzneimittel für seltene Leiden (Orphan Drug). Der Orphan-Drug-Status und der Break-through-Therapy-Status wurden auch von der US-amerikanischen Food and Drug Administration (FDA) zuerkannt. Mit seiner hohen Spezifität, Wirksamkeit und Verträglichkeit ist BLV ein sehr vielversprechendes Medikament zur Behandlung chronischer HBV- und insbesondere HDV-Infektionen.
